# Editorial

**DOI:** 10.1093/eurpub/ckac091

**Published:** 2022-08-29

This supplement is published ahead of the 11th conference of HEPA Europe, the WHO Regional Office for Europe network for the promotion of Health-Enhancing Physical Activity.

The HEPA Europe conference will focus on “An ecosystem approach to health-enhancing physical activity promotion”. The objective of the conference is to engage policymakers, professionals, scientists, and other stakeholders, as well as citizens to engage in the field of health-enhancing physical activity promotion.

The health benefits of physical activity, both in terms of reducing the risk of death and morbidity and in terms of well-being and quality of life, are now widely demonstrated and disseminated. The evidence confirming the benefits associated with physical activity has been accumulating over the last few years. Physical activity is now recognized as a major determinant of health and this recognition has resulted in its inclusion in the political agenda, at the national, European, and international levels. Public health can take the lead in bringing about the structural changes necessary to promote health-enhancing physical activity. The factors that support and hinder efforts to increase the physical activity levels of populations are complex and interconnected at many levels of influence, not always serving the interests of promoting health-enhancing physical activity. Physical activity must be considered at both the individual and collective levels, based on an ecosystem approach that recognizes that human populations are an integral component of many ecosystems. Thus, it is important to recognize that individual behaviors are the product of a complex interactions network, variations and changes, causes and influences. The ‘physical activity-health’ ecosystem is no exception to this, and any change in one of its component systems is likely to trigger a series of repercussions on each of the neighboring systems, which ultimately affect the level of physical activity of individuals and, directly or indirectly, their health. Physical activity has the strength and particularity to concern different sectors of activity (health, sport, transport, urbanism, social, environment, education, work etc.) and can potentially involve several sectors. Physical activity is one of the most effective ways to improve the health of populations and is a health determinant on which it is possible to act at different levels. It promotes a positive approach to health and its multi-sectoral nature is a lever to help address many different societal issues through coordinated policies for health-enhancing physical activity. The abstracts published in this supplement illustrate the diversity and the richness of physical activity for health. These abstracts invite the reader to engage in discussions on evidence-informed strategies, interventions, and tools to develop an ecosystem approach to health-enhancing physical activity promotion.

Anne Vuillemin, head of the Organizing Committee

Fabienne d’Arripe-Longueville, on behalf of the Scientific Committee and Kremlin Wickramasinghe, Regional Advisor Nutrition, Physical Activity and Obesity, WHO Regional Office for Europe.

